# Evidence of a new metabolic capacity in an emerging diarrheal pathogen: lessons from the draft genomes of *Vibrio fluvialis* strains PG41 and I21563

**DOI:** 10.1186/1757-4749-5-20

**Published:** 2013-07-29

**Authors:** Indu Khatri, Sakshi Mahajan, Chetna Dureja, Srikrishna Subramanian, Saumya Raychaudhuri

**Affiliations:** 1 CSIR-Institute of Microbial Technology, Chandigarh, India

**Keywords:** Metabolic fitness, *Eut*-operon, Vibrionaceae

## Abstract

**Background:**

*Vibrio fluvialis* is an emerging diarrheal pathogen for which no genome is currently available. In this work, draft genomes of two closely related clinical strains PG41 and I21563 have been explored.

**Results:**

*V. fluvialis* strains PG41 and I21563 were sequenced on the Illumina HiSeq 1000 platform to obtain draft genomes of 5.3 Mbp and 4.4 Mbp respectively. Our genome data reveal the presence of genes involved in ethanolamine utilization, which is further experimentally confirmed by growth analysis.

**Conclusions:**

Combined *in silico* and growth analysis establish a new metabolic capacity of *V. fluvialis* to harvest energy from ethanolamine.

## Background

The genus *Vibrio* of the class *Gammaproteobacteria* is an ecologically and metabolically diverse group autochthonous to the marine, estuarine, and freshwater environment [1]. This genus comprises of nearly 100 species of which, some members are capable of causing severe diarrheal diseases, thus posing a serious threat in the developing world [2,3]. Among these, *Vibrio cholerae* O1/O139 and *Vibrio parahaemolyticus* are considered major diarrheal pathogens and are responsible for several pandemics and epidemics [4,5]. The other members of the *Vibrionaceae* family namely *Vibrio mimicus* and *Vibirio fluvialis* are also frequently found to be associated in diarrheal outbreaks [6,7].

*Vibrio fluvialis* is a halophilic, polarly-flagellated, Gram-negative bacterium. It was first isolated in 1975 from the stool of a diarrhea patient in Bahrain and categorized as group F *Vibrio* and rechristened as *Vibrio fluvialis* in 1981 [8]. Since its discovery, the organism has been implicated in several outbreaks and sporadic cases of diarrhea [9]. Between 1976 and 1977, 500 patients (mostly children and young adults) were reported to be infected with *Vibrio fluvialis* in Bangladesh with symptoms marked by vomiting, abdominal pain, moderate to severe dehydration and significant fever [10]. In the United States, *Vibrio fluvialis* has been associated with enterocolitis in infants [11]. In Indonesia, *Vibrio fluvialis* has been recognized as one of major enteric pathogen causing cholera-like diarrhea [12]. Recently, an examination of 400 non-agglutinating *Vibrio* species collected from patients with diarrhea in the period 2002-2009 in Kolkata, India identified 131 strains of *Vibrio fluvialis* of which 43 strains were suggested to be the sole pathogen and the remaining 88 strains were co-pathogens with other prominent enteric pathogens [7]. In 2009, an episode of massive diarrhea broke out in coastal regions of India following the cyclone Aila. Further investigation confirmed *Vibrio fluvialis* as the predominant pathogen responsible for this diarrheal outbreak [13]. Clinically, *Vibrio fluvialis* causes diarrhea having symptoms similar to that of cholera [14]. The organism contains El Tor-like hemolysin [15] and exhibits cytotoxic and cell-vacuolating activity on HeLa cells [16]. Collectively, the information garnered from epidemiological studies clearly establishes *Vibrio fluvialis* as an emerging diarrheal pathogen. The situation is further aggravated by the characterization of several multi-drug resistant clinical isolates of this strain [17,18].

There is now a growing realization regarding the significance of ethanolamine (EA), a small molecule present abundantly in host diet, as well as in bacterial and epithelial cells of the vertebrate intestine, that acts as an energy source for numerous bacteria including pathogens [19]. Using *Salmonella enterica* serovar Typhimurium as a model organism, the process of utilization of EA as an energy source has been demonstrated previously. The *eut* operon contains 17 genes whose concerted action converts EA into more metabolically suitable molecules. In case of *Salmonella enterica*, all essential proteins for EA metabolism are clustered into a multiprotein complex known as the metabolosome, which is reminiscent of the bacterial micro-compartment [20]. The presence of ethanolamine lyase (EutBC), a key enzyme of EA utilization machinery (*eut*) has been established in about 100 bacterial genomes [21]. In a recent effort, our group has uncovered the presence of *eut* operon in *Vibrio alginolyticus* and the capacity of this bacterium to utilize EA as a nitrogen source [22].

Taxonomically, *Vibrio fluvialis* belongs to the Cholerae clade. The other members of the Cholerae clade are *Vibrio furnissii*, *Vibrio cholerae*, *Vibrio mimicus* and *Vibrio metschnikovii*[23]. Genomic analysis of the members of the Cholerae clade reveals the presence of *eut* operon genes, thus indicating the possibility of such metabolic potential in these bacteria [22]. So far, no genome information for any strain of *Vibrio fluvialis* is available. This prompted us to embark on the present study to decipher the genome and examine the ability of *Vibrio fluvialis* to harvest energy from EA.

## Methods

### Genome sequencing

To pursue our interest, two *Vibrio fluvialis* strains namely PG41 and I21563, clinical isolates of 1998 and 2004 outbreaks respectively [5,7] were sequenced using the Illumina-HiSeq 1000 technology (See Additional file 1). For genome analysis, library preparation was carried out according to the Tru Seq DNA sample preparation protocol (Illumina, Inc., San Diego, CA) at C-CAMP, Bangalore, India. Briefly, 1 μg of bacterial DNA was sheared to an average length of 300 to 400 bp, and standard blunt ending with “A” base (paired-end DNA sample preparation kit; Illumina, Inc.) was performed. Illumina index adapters were ligated to the ends of the fragments. After ligation reaction and separation of non-ligated adapters, samples were amplified by PCR for 8 cycles to selectively enrich those fragments in the library having adapter molecules at both ends. The sample was quantified and the quality was tested using a Bioanalyzer. Libraries were sequenced in a paired-end 100 base run, using TruSeq PE Cluster Kit v3-cBot-HS for cluster generation on C-bot and TruSeq SBS Kit v3-HS for sequencing on the Illumina HiSeq1000 platform according to manufacturer recommended protocols. A total of 24,420,454 and 21,454,382 paired-end reads were obtained for *V. fluvialis* strains PG41 and I21563, respectively.

### Assembly and annotation

*De novo* assembly approach was used to finalize the draft genomes using CLCbio wb6. The genomes were assembled with several different parameters. The genome finishing module of CLCbio was applied on the best assembly. The contigs thus obtained were scaffolded using SSPACE v2.0 scaffolder [24] and the gaps were filled by GapFiller v1.10 [25]. The gap-filled scaffolds thus obtained, were broken at the unfilled gaps. Functional annotation was carried out by RAST (Rapid Annotation using Subsystem Technology) [26], tRNA was predicted by tRNAscan-SE 1.23 [27] and rRNA genes by RNAmmer 1.2 [28].

### Submission of genome sequence

This Whole Genome Shotgun project has been deposited at DDBJ/EMBL/GenBank under the accession ASXS00000000 and ASXT00000000 for *Vibrio fluvialis* PG41 and *Vibrio fluvialis* I21563 respectively. The version described in this paper is the first version ASXS01000000 and ASXT01000000.

## Quality assurance

**Figure 1:**
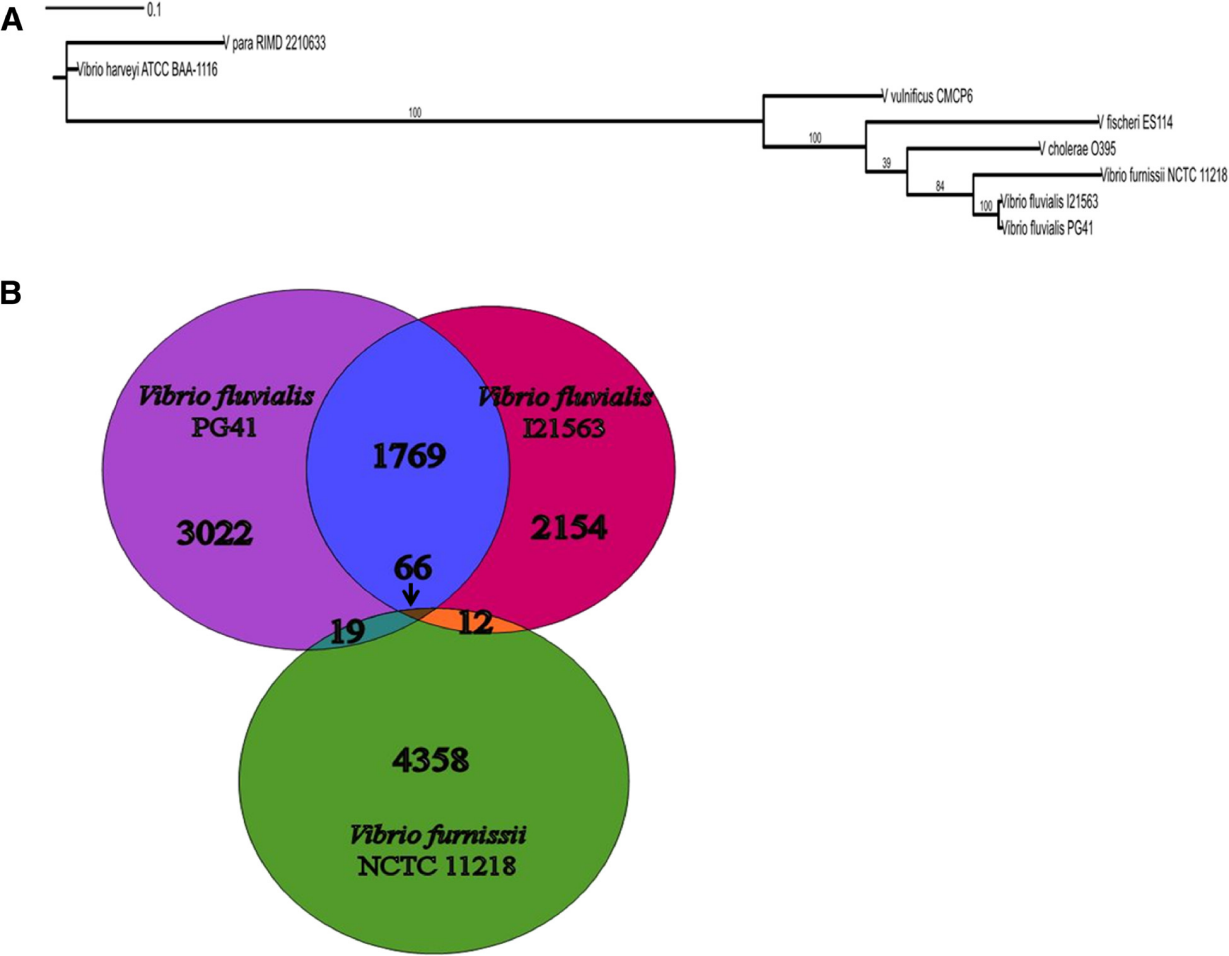
***Vibrio fluvialis *phylogeny and proteome comparison. (A)** MLSA tree of six housekeeping genes for species characterization; **(B)** Proteome similarity between *Vibrio fluvialis* PG41, *Vibrio fluvialis* I21563 and *Vibrio furnissii* NCTC 11218.

The genomic DNA was isolated from pure bacterial isolate and was further confirmed by 16S rDNA gene sequencing (See Additional file 1) as well as examining certain phenotypic characteristics such as tolerance to high salt and negative for gas production in glucose-rich media which are defining characteristics of *Vibrio fluvialis*[16]. Multi Locus Sequence Analysis (MLSA) tree was generated with the gene sequences of six housekeeping genes *ftsZ*, *mreB*, *pyrH*, *recA*, *rpoA* and *topA* for species characterization and confirmation. The concatenated sequences for these genes were aligned using PCMA [29]. PhyML tree was build using Topali v2.5 [30] (HKY model, 100 bootstraps) (Figure 1A). The proteins of *Vibrio fluvialis* strain PG41 was subjected to BLASTp at E-value 1e^-5^ to *Vibrio fluvialis* strain I21563 to find the proteome similarity percentage between the two strains.

## Results and discussion

### Genome characteristics

Genome of *V. fluvialis* PG41 and *V. fluvialis* I21563 was sequenced on Illumina HiSeq 1000 technology that resulted in a total of 24,420,454 and 21,454,382 paired-end reads of length 101 bp. A total of 24,227,857 high-quality reads for *V. fluvialis* PG41 with approximately 450x coverage were assembled with CLCbio wb6 (parameters: word size 55 and bubble size 65) to obtain a draft genome of 5.3 Mbp in 72 contigs (N50: 229,236 bp). Likewise, 21,175,217 high-quality reads from *V. fluvialis* I21563 with approximately 490× coverage were assembled with CLCbio wb6 (parameters: word size 40 and bubble size 50) to obtain a draft genome of 4.4 Mbp in 92 contigs (N50: 104,410 bp). The genome finishing module of CLCbio followed by SSPACE v2.0 scaffolder and GapFiller v1.10 were used. The gap-filled scaffolds thus obtained, were broken at the unfilled gaps to obtain 49 contigs (N50: 236,090 bp; GC content: 48%) of 5,343,550 bp for *V. fluvialis* PG41 and 84 contigs (N50: 109,625 bp; GC content: 50%) of 4,371,313 bp for *V. fluvialis* I21563. The genomes contain 3 rRNA genes (5S-23S-16S) in both strains of *V. fluvialis* and 126 and 72 aminoacyl-tRNA synthetase genes in *V. fluvialis* PG41 and *V. fluvialis* I21563, respectively. A total of 4856 coding regions were found in *V. fluvialis* PG41 genome, of which 3527 (72%) could be functionally annotated whereas 3990 coding regions were identified in the genome of *V. fluvialis* strain I21563, of which 3263 (82%) could be functionally annotated. Both strains of *V. fluvialis*, despite their differences in genome size and the total number of predicted proteins, have 1835 proteins in common at 100% identity. Of these 1835 proteins, 66 proteins have 100% identity to the proteins of *Vibrio furnissii* NCTC 11218 (Figure 1B). RAST server based annotation of the whole genome, revealed the presence of 505 subsystems in *Vibrio fluvialis* PG41 and 493 subsystems in *Vibrio fluvialis* I21563 (Table 1).

**Table 1:** **Comparison of subsystem features between the draft genomes of *Vibrio fluvialis *PG41 and *Vibrio fluvialis *I21563**

**Subsystem features**	**CDS present**	
	***Vibrio fluvialis *I21563**	***Vibrio fluvialis *PG41**
**Cofactors, Vitamins, Prosthetic Groups, Pigments**	244	267
**Cell Wall and Capsule**	112	178
**Virulence, Disease and Defense**	93	84
**Potassium metabolism**	21	21
**Miscellaneous**	33	33
**Phages, Prophages, Transposable elements, Plasmids**	10	15
**Membrane Transport**	164	180
**Iron acquisition and metabolism**	47	51
**RNA Metabolism**	149	113
**Nucleosides and Nucleotides**	93	114
**Protein Metabolism**	170	214
**Cell Division and Cell Cycle**	30	28
**Motility and Chemotaxis**	160	166
**Regulation and Cell signaling**	91	95
**Secondary Metabolism**	4	4
**DNA Metabolism**	133	136
**Regulons**	9	9
**Fatty Acids, Lipids, and Isoprenoids**	114	120
**Nitrogen Metabolism**	49	52
**Dormancy and Sporulation**	3	5
**Respiration**	127	129
**Stress Response**	121	133
**Metabolism of Aromatic Compounds**	17	17
**Amino Acids and Derivatives**	425	444
**Sulfur Metabolism**	31	32
**Phosphorus Metabolism**	56	56
**Carbohydrates**	427	494

### Existence of *eut*-operon

It has been shown that EA, a small host derived molecule serves as an energy source for many bacteria including pathogens such as *Salmonella enterica* serotype *Typhimurium* and Enterohaemorrhagic *Escherichia coli* (EHEC) [19,20,31,32]. Recently, our group has established the potential of EA utilization in *Vibrio alginolyticus*[23]. We therefore examined the presence of genes related to EA utilization pathway in the genomes of the *Vibrio fluvialis* strains and compared it to homologs from *Vibrio alginolyticus*. It has been documented that genes from the EA utilization machinery can be clustered in the form of short or long operons [21]. The genomes of both *Vibrio fluvialis* strains have the short operon. Only EutBCEGPR and ethanolamine permease proteins could be identified in the draft genomes. Genes corresponding to EutRBC and ethanolamine permease are in genome context (Table 2). The percentage identity was evaluated for Eut proteins in *V. alginolyticus* 12G01 and *Vibrio fluvialis* (Table 1). EutBCEG of both the *Vibrio fluvialis* strains are > 50% identical to the respective proteins in *Vibrio alginolyticus* 12G01. EutPR and ethanolamine permease are ~30% identical to the proteins of *Vibrio alginolyticus*. We could not find any homologs of the *eutD* and *eutQ* genes in the draft genomes of *Vibrio fluvialis* strains PG41 and I21563. The *eut* operon is 100% conserved between the *Vibrio fluvialis* strains PG41 and I21563 and share more than 90% sequence identity to their homologs in *Vibrio furnissii* NCTC 11218, the closest *Vibrio* for which whole genome information is available.

**Table 2:** **Genomic positions of ethanolamine utilization proteins in *Vibrio fluvialis *PG41 and *Vibrio fluvialis *I21563**

	***Vibrio fluvialis *PG41**				***Vibrio fluvialis *I21563**			
**Eut proteins**	**Locus_tag**	**Contig**	**Start**	**End**	**Locus_tag**	**Contig**	**Start**	**End**
**EutG**	L910_2070	2	57060	58208	L911_1325	21	57070	58218
**EutR**	L910_3169	3	301625	300792	L911_0583	14	121312	120479
**EutB**	L910_3170	3	302044	303447	L911_0584	14	121731	123134
**EutC**	L910_3171	3	303444	304286	L911_0585	14	123131	123973
**Ethanolamine permease**	L910_3172	3	304317	305711	L911_0586	14	124004	125398
**EutE**	L910_4642	9	2285	4987	L911_3405	50	27410	24708
**EutP**	L910_1582	15	173979	175775	L911_2069	30	21335	19671

### *Vibrio fluvialis* utilizes ethanolamine as an energy source

**Figure 2:**
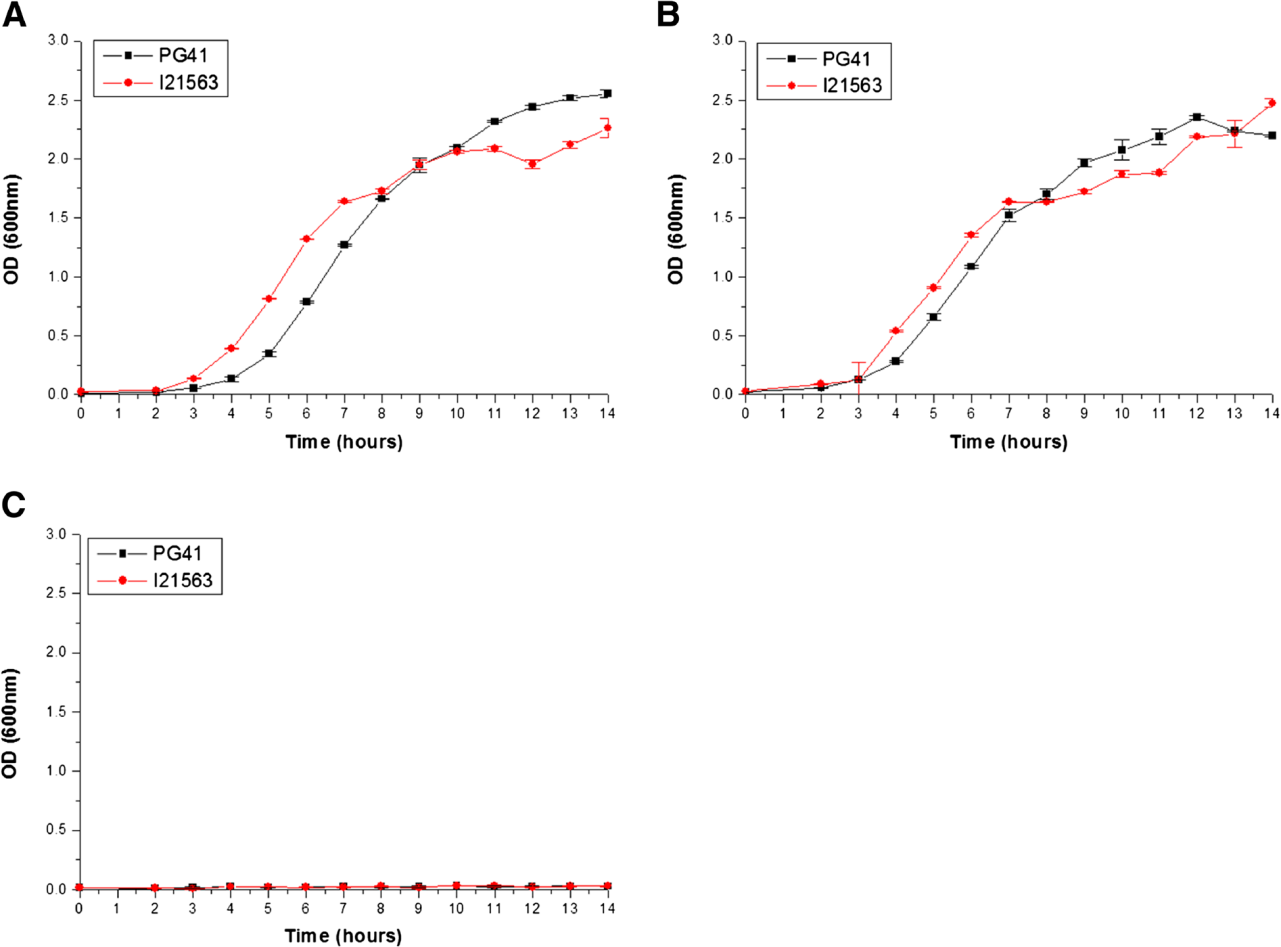
**Growth curves of PG41 and I21563. (A)** Minimal media complete; **(B)** Minimal media containing ethanolamine as a nitrogen source; **(C)** Minimal media containing ethanolamine as a carbon source. OD (600 nm) of two biological replicates was tracked for 14 hours. Error bars depict the standard deviation from the mean.

As evident from the preceding section, the genome of *Vibrio fluvialis* strains contain genes encoding proteins of the *eut* operon (Table 3). To ascertain the capacity of *V. fluvialis* to utilize EA as an energy source, two clinical isolates of *Vibrio fluvialis viz.*, PG41 and I21563 were subjected to growth analysis. The growth experiment was carried out in minimal media supplemented with EA as an energy source using a previously described procedure [22,31]. Briefly, overnight Luria broth grown cultures of these strains were further diluted and grown to bacterial OD of 1.0 at 37°C in Luria broth. The cultures were centrifuged, washed and again diluted 100-fold in M9 minimal salt medium containing KH_2_PO_4_ (15 g l^-1^), Na_2_PO_4_.7H_2_O (64 g l^-1^), NaCl (2.5 g l^-1^) supplemented with 0.1 mM CaCl_2_, 2 mM MgSO_4,_ and 200 nM vitamin B12 (cyanocobalamin). To evaluate the ability of *Vibrio fluvialis* strains to utilize EA as a nitrogen source, 82 mM of ethanolamine hydrochloride was added along with 0.4% glucose to minimal medium. To test the ability of these strains to use EA as a carbon source, 82 mM of ethanolamine hydrochloride and NH_4_Cl (5.0 g l^-1^) were added to minimal medium. Cultures were then incubated with agitation (200 rpm) at 37°C and growth was monitored over 14 h. Our data suggests that both *Vibrio fluvialis* strains are capable of utilizing EA as a nitrogen source rather than a carbon source. This finding is in agreement with a similar study reported earlier for *Vibrio alginolyticus*[22] (Figure 2). Interestingly, *Salmonella* utilizes EA both as a nitrogen and carbon source [19], while EHEC and *Vibrio alginolyticus* prefers to use it as a nitrogen source [22,31].

**Table 3:** **Percentage identity of ethanolamine utilization proteins of *Vibrio fluvialis *strains PG41 and I21563 with *Vibrio alginolyticus *12G01**

***Vibrio alginolyticus *12G01**	***Vibrio fluvialis *PG41**	***Vibrio fluvialis *I21563**
**(*eut *operon proteins)**	**(% identity)**	**(% identity)**
**EutB**	78	78
**EutC**	57	57
**EutE**	92	92
**EutG**	74	74
**EutP**	33	33
**EutR**	36	36
**eat**	37	37

## Future directions

Compared to other notable diarrheal pathogens of the *Vibrionaceae* family, our understanding of the biology of *Vibrio fluvialis* is not sufficiently explored. Recent works have highlighted some information on the epidemiology and pathogenic determinants of *Vibrio fluvialis*. In this regard, our draft genomes will serve as a good starting point to explore and obtain novel insights into the biology of this emerging diarrheal pathogen. Moreover, regulation of *eut* operon and significance of EA in controlling virulence as seen in other pathogens could be examined in *Vibrio fluvialis* and this is likely to shed additional light on the pathogenesis and ecology of this emerging pathogen.

## Availability of supporting data

This Whole Genome Shotgun project has been deposited at DDBJ/EMBL/GenBank under the accession ASXS00000000 and ASXT00000000 for *Vibrio fluvialis* PG41 and *Vibrio fluvialis* I21563 respectively. The version described in this paper is the first version ASXS01000000 and ASXT01000000.

## Conclusions

Our draft genome analysis clearly reveals the existence of the *eut* machinery in *Vibrio fluvialis*, thereby highlighting a new metabolic potential of this bacterium. Furthermore, growth analysis clearly demonstrates the capacity of this organism to harvest energy from EA preferably as a nitrogen source.

## Abbreviations

PCMA: Profile Consistency Multiple Sequence Alignment; HKY: Hasegawa, Kishino and Yano; RAST: Rapid Annotation using Subsystem Technology; EA: Ethanolamine; EHEC: Enterohaemorrhagic *Escherichia coli*; MLSA: Multi Locus Sequence Analysis.

## Competing interest

The authors declare that they have no competing interests.

## Authors’ contributions

SRC conceived the idea; SM and CD isolated chromosomal DNA and carried out strain identification, 16S rDNA sequencing and growth analysis of *Vibrio fluvialis* strains; IK and SS carried out the assembly, annotation and analysis of the genomic data. IK and SRC wrote the manuscript. All authors have read and approved the manuscript.

## Supplementary Material

Additional file 1 Materials and methods. Click here for file
