# Supplementary material for: Evidence of a new metabolic capacity in an emerging diarrheal pathogen: lessons from the draft genomes of Vibrio fluvialis strains PG41 and I21563
Source: Gut Pathog. 2013 Jul 29;5:20. doi: 10.1186/1757-4749-5-20 (PMC3729366; doi:10.1186/1757-4749-5-20)
Supplement: Additional file 1 — Materials and methods. [file 1757-4749-5-20-S1.pdf]

Bacterial strains and preservation

The clinical isolates of *V. fluvialis* were procured from NICED, Calcutta. The strains were propagated at 37°C in liquid with agitation or on solid (1.5% agar) in Luria broth unless mentioned otherwise. For long term storage, organisms were maintained in Luria Bertani (Difco Laboratories) with 15% glycerol at −80°C.

Genomic DNA isolation

DNA was isolated from overnight cultures of *Vibrio fluvialis* grown in Luria broth at 37°C with agitation by the method of Mekalanos, 1983, with minor modifications. Briefly, the cells from 1.5 ml of culture were resuspended in 0.6 ml of 10 mM Tris-HCI (pH 8.0), 1 mM EDTA and 20 µg/ml RNaseA. To the resuspended cells, 0.6% (w/v) of SDS and 3 µl of 20 mg/ml proteinase K were added. After mixing, the cell lysate was incubated at 37°C for 1 hr. After incubation, 100 µl of 5 M NaCl was added followed by addition of 80 µl of 10% CTAB. The solution was incubated at 65°C for 10 minutes. DNA was then extracted with 0.6 ml chloroform:isoamyl alcohol (24:1). The aqueous phase was collected into a new tube and the nucleic acids were precipitated by addition of 0.6 volume of isopropanol. The DNA was washed with 70% ethanol twice. The DNA was then dried and dissolved in 10 mM Tris-HCI (pH 7.4) and 1 mM EDTA and stored at 4°C.

Amplification

The PCR amplification were carried with 50 to 100 ng of template DNA in a final reaction volume of 50 µl containing 200 µM of each dNTP, 100ng of each primer, 1X PCR buffer and 1.5 units of Taq DNA polymerase. PCR conditions were as follows: a single step of denaturation of template DNA at 94 °C for 1 min, followed by amplification of 30 cycles, each of which consisted of three steps, denaturation at 94 °C for 30 s, primer annealing at 55–60 °C (depending upon the primers used) for 30 s, and primer extension at 72 °C for a time period based on the expected size of the amplicon (usually 1 min per kb of DNA fragment), followed by a single step of final extension at 72 °C for 7 min. PCR product was then analyzed on 1% agarose gel. PCR products were purified using Qiaquick PCR purification kit.

Library preparation and sequencing

The library preparation was carried out according to the Tru Seq DNA sample preparation protocol (Illumina, Inc., San Diego, CA) at C-CAMP, Bangalore, India. 1 µg of bacterial DNA was sheared to an average length of 300 to 400 bp. End repair, A-tailing and adapter ligation (~120 base adapter) was performed according to paired-end DNA sample preparation kit (Illumina, Inc.). Size selection of adapter ligated DNA was done in range of 400 to 550 bases for DNA library. The insert size was taken in range of 280 to 430 bases for DNA library. PCR enrichment was performed for 8 cycles and the samples were validated on a bioanalyzer. Libraries were sequenced in a Paired End 100 base run, using TruSeq PE Cluster Kit v3-cBot-HS for cluster generation on C-bot and TruSeq SBS Kit v3-HS (Catalog No.: PE-401-3001) for sequencing on the Illumina HiSeq 1000 platform according to recommended protocols.

Read Quality Assessment

Read quality was assessed by FastQC software (http://www.bioinformatics.babraham.ac.uk/projects/fastqc). All reads were 101 bp in length and there were no overrepresented sequences and Kmers. Quality score across all bases is based on Illumina 1.9 encoding and average quality per read is 37 for *Vibrio fluvialis* PG41 and 36 for *Vibrio fluvialis* I21563 reads. GC content is below 50%.
